# Memory-built-in quantum cloning in a hybrid solid-state spin register

**DOI:** 10.1038/srep12203

**Published:** 2015-07-16

**Authors:** W.-B. Wang, C. Zu, L. He, W.-G. Zhang, L.-M. Duan

**Affiliations:** 1 Center for Quantum Information, IIIS, Tsinghua University, Beijing 100084, PR China; 2 Department of Physics, University of Michigan, Ann Arbor, Michigan 48109, USA

## Abstract

As a way to circumvent the quantum no-cloning theorem, approximate quantum cloning protocols have received wide attention with remarkable applications. Copying of quantum states to memory qubits provides an important strategy for eavesdropping in quantum cryptography. We report an experiment that realizes cloning of quantum states from an electron spin to a nuclear spin in a hybrid solid-state spin register with near-optimal fidelity. The nuclear spin provides an ideal memory qubit at room temperature, which stores the cloned quantum states for a millisecond under ambient conditions, exceeding the lifetime of the original quantum state carried by the electron spin by orders of magnitude. The realization of a cloning machine with built-in quantum memory provides a key step for application of quantum cloning in quantum information science.

As a basic property of quantum mechanics, quantum no-cloning theorem states that it is impossible to perfectly clone unknown quantum states^1^,^2^, which has far-reaching implications in quantum information science^3^,^4^. The security of quantum cryptography^5^, the privacy of quantum computation^6^, and the complexity of quantum error correction^7^, at the fundamental level, are all related to this theorem. Given the quantum no-cloning theorem, a natural question, as first raised by Buzek and Hillery^8^, is what are the best cloning machines allowed by quantum mechanics. The study of optimal cloning machines attracts strong interest^9^,^10^,^11^,^12^,^13^,^14^,^15^,^16^,^17^,^18^,^19^,^20^,^21^, and the approximate cloning protocols have found important applications in quantum information science^3^,^5^. For instance, one of the best eavesdropping strategies for quantum cryptography is to make an approximate copy of the quantum state sent by Alice (the message sender), store it in a quantum memory, and make an optimal measurement on the cloned state after the communication between Alice and Bob (the message receiver)^3^,^5^.

On the experimental side, approximate quantum cloning of states has been demonstrated from a photon to another photon, using either stimulated emission^13^,^14^ or Hong-Ou-Mandel filtering^15^,^16^,^17^. The cloning process in those experiments is intrinsically probabilistic due to post-selection of the desired sate components by the photon detection^13^,^14^,^15^,^16^,^17^. Other demonstrations have also been reported^18^,^19^,^20^,^21^, however, they are limited to copying or transferring quantum states within the Hilbert space of a single particle^18^,^19^, or based on a thermal ensemble of nuclear magnetic resonance (NMR) system in a highly mixed state which does not constitute true cloning of individual quantum particles^20^,^21^. To make quantum cloning useful, it is important to have the ability to deterministically copy quantum states from one particle to another, which is yet to be demonstrated.

In this paper, we report an experiment that achieves cloning of quantum states from the electron spin to the nuclear spin in a hybrid solid-state spin register. The cloned state and the original state are carried by different types of particles in a diamond sample. Our experiment is based on control of an individual nitrogen vacancy (NV) center in the diamond. The NV center is a diamond defect that attracts strong interest in recent years with great potential for implementation of quantum information protocols^22^,^23^,^24^,^25^,^26^,^27^,^28^,^29^,^30^,^31^,^32^. An individual NV center makes a hybrid spin register consisting of the electron spin and the nuclear spins in the proximity. The nuclear spin is a natural memory qubit which can store the cloned quantum state^23^,^27^, while the electron spin that carries the original state can be linked with photons, the ideal flying qubits for quantum communication, through a quantum interface^23^,^28^,^29^. These features are important for application of quantum cloning^3^,^5^. The cloning operation in our experiment is achieved through quantum gates and deterministic by itself. However, to verify quantum cloning, we also need initial state preparation and final state detection, which is not deterministic yet and requires ensemble averaging in our experiment. Deterministic initialization and single-shot readout could be achieved for the NV center system at the cryogenic temperature or by shining laser beams at specific wavelengths^33^. We have observed a storage time of the cloned quantum state in the nuclear spin for about a millisecond under ambient conditions, which exceeds the coherence time of the electron spin under free evolution by several orders of magnitude. With isotopically purified diamond samples, the coherence time of the nuclear spin can be pushed up to seconds^27^ or probably even hours at room temperature^23^, making it an ideal memory qubit.

## Results

### Experimental deterministic quantum cloning operation

**Figure 1:**
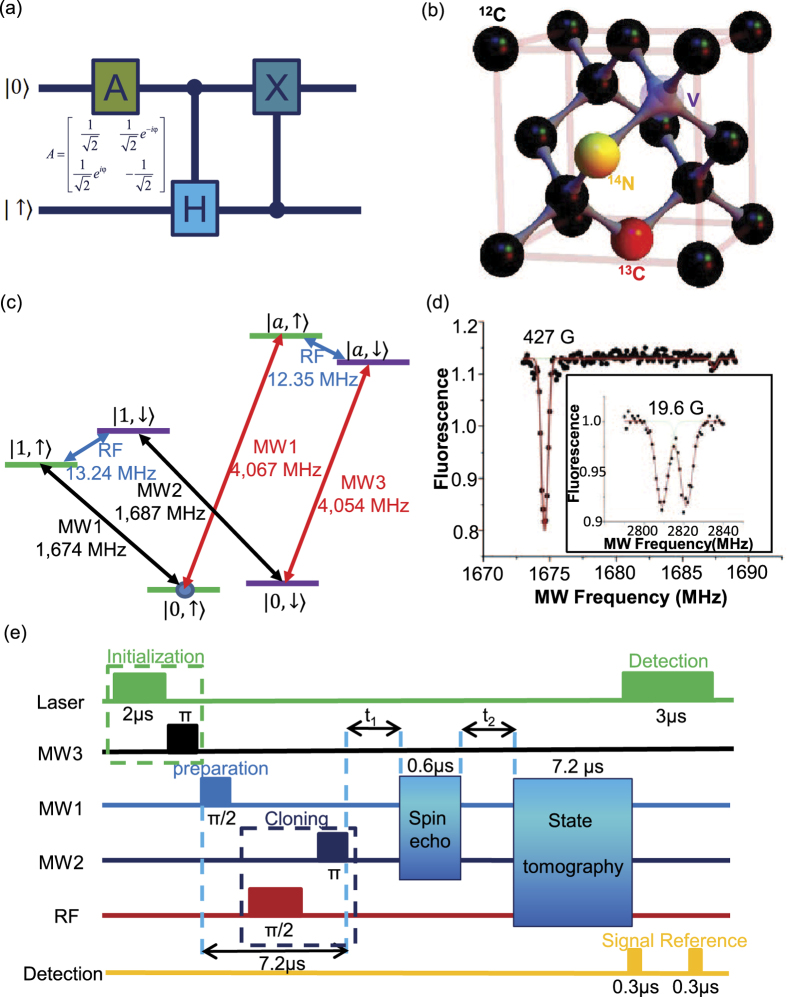
The setup for implementation of quantum cloning operation. (**a**) The quantum circuit for phase-covariant quantum cloning, where *A* denotes the unitary to prepare the input state on the equator of the Bloch sphere. (**b**) Illustration of a nitrogen vacancy (NV) center in a diamond with a proximal *C*^13^ atom. The electron spin of the NV and the nuclear spin of the *C*^13^ atom are used for implementation of quantum cloning. (**c**) The level structure of the electron spin and the nuclear spin, which includes contribution of the Zeeman energy, the intrinsic splitting of the NV spin levels, and the hyperfine interaction from both the *C*^13^ and the host *N*^14^ nuclear spins (the *N*^14^ nuclear spin is polarized to the |+1〉 state by the initial optical pumping and contributes a fixed shift about 2.16 *MHz* to the level splitting). Different Zeeman levels of the electron spin are coupled through the microwave transitions |0〉 ↔ |1〉 and |0〉 ↔ |a〉. The nuclear Zeeman levels are coupled through the r.f. transition |↑〉 ↔ |↓〉. (**d**) The optically detected magnetic resonance (ODMR) spectroscopy obtained by measuring the fluorescence level when scanning the frequency of the microwave field that couples to the electron spin |0〉 to |1〉 transition. The two symmetric dips at 19.6 G magnetic field shown in the insert represent the hyperfine splitting caused by the unpolarized nuclear spin. The corresponding asymmetric dips at 427 G field indicates that the nuclear spin has been polarized. (**e**) The time sequences of the laser, microwave, and r.f. pulses and the detector open windows for the spin initialization, the input state preparation, the cloning operation, the spin echo, and the final state verification through the quantum state tomography.

For optimal approximate quantum cloning, we start with an unknown quantum state |Ψ〉 of particle 1 and a fixed blank state |0〉 of particle 2, and transform the initial state |Ψ〉_1_⊗|0〉_2_ to a two-particle entangled state |Φ〉_12_ through a unitary operation *U* so that the reduced state *ρ*_1_ = *tr*_2_(|Φ〉_12_〈Φ|) and *ρ*_2_ = *tr*_1_(|Φ〉_12_〈Φ|) best approximate the state |Ψ〉 with a maximized fidelity *F* = 〈Ψ|*ρ*_1_|Ψ〉 = 〈Ψ|*ρ*_2_|Ψ〉. We consider in this paper the phase-covariant quantum cloning where the state |Ψ〉 is randomly taken from the qubit states 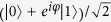 on the equator of the Bloch sphere with an unknown phase *φ*^3^,^12^. This type of cloning is most relevant for application in quantum cryptography as the well-known BB84 quantum key distribution protocol is based on transmission of four randomly chosen qubit states on the Bloch-sphere equator with the phase *ϕ* = 0, *π*/2, *π*, 3*π*/2, respectively^3^,^5^. For the phase-covariant quantum cloning, the unitary operation *U* can be realized through a quantum circuit *CH*_12_*CNOT*_21_ shown in Fig. 1(a), where *CH*_12_ and *CNOT*_21_ represent the controlled-Hadamard and the controlled-NOT gates, respectively, with the first subscript as the control qubit and the second as the target. The final state after cloning takes the form 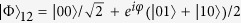, with the optimal fidelity 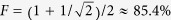.

In our experimental realization of quantum cloning, the original state is carried by the electron spin state of a diamond NV center. The NV center has spin-triplet electronic ground state with the Zeeman levels |*m*_*s*_ = 0, ± 1〉. We use |0〉 ≡ |*m*_*s*_ = 0〉 and |1〉 ≡ |*m*_*s*_ = −1〉 as the qubit basis-vectors to encode the original state |Ψ〉 and |*a*〉 ≡ |*m*_*s*_ = 1〉 as an ancillary level. The state |Ψ〉 is cloned from the electron spin to a proximal ^13^*C* nuclear spin shown in Fig. 1(b) with the nuclear Zeeman levels denoted by |↑〉 and |↓〉. The complete level structure and the associated energy splittings are shown in Fig. 1(c). Our experimental setup is described in detail in Ref. 30. Microwave fields couple the electron spin from the state |0〉 to |1〉 or |*a*〉, and a radio-frequency (r.f.) field drives the nuclear spin transition between |↑〉 and |↓〉. Due to the hyperfine interaction between the electron and the nuclear spins, the transitions between different levels shown in Fig. 1(c) have different resonance frequencies, which can be selectively driven by the microwave or r.f. fields, leading to controlled gate operations between the electron and the nuclear qubits.

To perform and verify quantum cloning operation, we need to apply a sequence of laser, microwave, and r.f. pulses as shown in Fig. 1(d) for initial state preparation, cloning, and final state detection. The electron spin is initialized through optical pumping to the level |*m*_*s*_ = 0〉 by illumination of a green laser for 2 *μs*^22^,^34^. The NV center has about 30% probability in the charge neutral (NV^0^) state under initialization by the a green laser^35^, which makes the state initialization step non-deterministic. With an external magnetic field of 427 G applied along the NV center axis, the same laser illumination also polarizes the nuclear spin to the level |↑〉 through the electron spin nuclear spin flip-flop process at the optically excited states^34^. The polarization of the nuclear spin is confirmed through the optically detected magnetic resonance (ODMR) shown in Fig. 1(d). The optical pumping prepares the system dominantly in the state |0, ↑〉, with a small fraction of population in |0, ↓〉 due to imperfection of the nuclear spin polarization. We then apply a microwave *π*-pulse to the transition |0, ↓〉 ↔ |*a*, ↓〉, transferring the small population from |0, ↓〉 to a shelter level |*a*, ↓〉. The level |a, ↓〉 is outside of the qubit space and its population thus has no influence on the experimental detection.

After initialization to the level |0, ↑〉, the electron spin is prepared to the input state 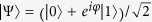 on the equator of the Bloch sphere through a microwave *π*/2-pulse with a tunable phase φ. We then perform quantum cloning by applying two conditional gates: A r.f. *π*/2-pulse applied to the transition |1, ↑〉 ↔ |1, ↓〉 induces a controlled Hadamard operation *CH*_12_, which transforms the state from |Ψ〉⊗|↑〉 to 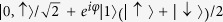. A microwave *π*-pulse resonant with the transition |1, ↓〉 ↔ |0, ↓〉 induces a CNOT gate *CNOT*_21_, transferring the state to the final form 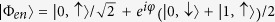. Note that the cloning operation is independent of the unknown phase *φ*. Due to the small magnetic moment of the nuclear spin, the gate *CH*_12_ by the r.f. pulse is slow with the gate time longer than the electron spin coherence time (about 1.7 *μs* under free evolution). To overcome the influence of dephasing of the electron spin caused by the nuclear spin environment, we apply dynamical decoupling to recover the electron spin coherence at the point of detection^36^. The simplest dynamical decoupling sequence is made of just a Hahn spin echo pulse applied after the cloning operation. With an appropriate choice of the delay time after the echo pulse to detect each component of the final experimental density matrix *ρ*_*en*_ of the electron and the nuclear spins, we can remove the influence of electron dephasing on the experimental verification of quantum cloning operation (see supplementary information).

### Verification of quantum cloning operation

**Figure 2:**
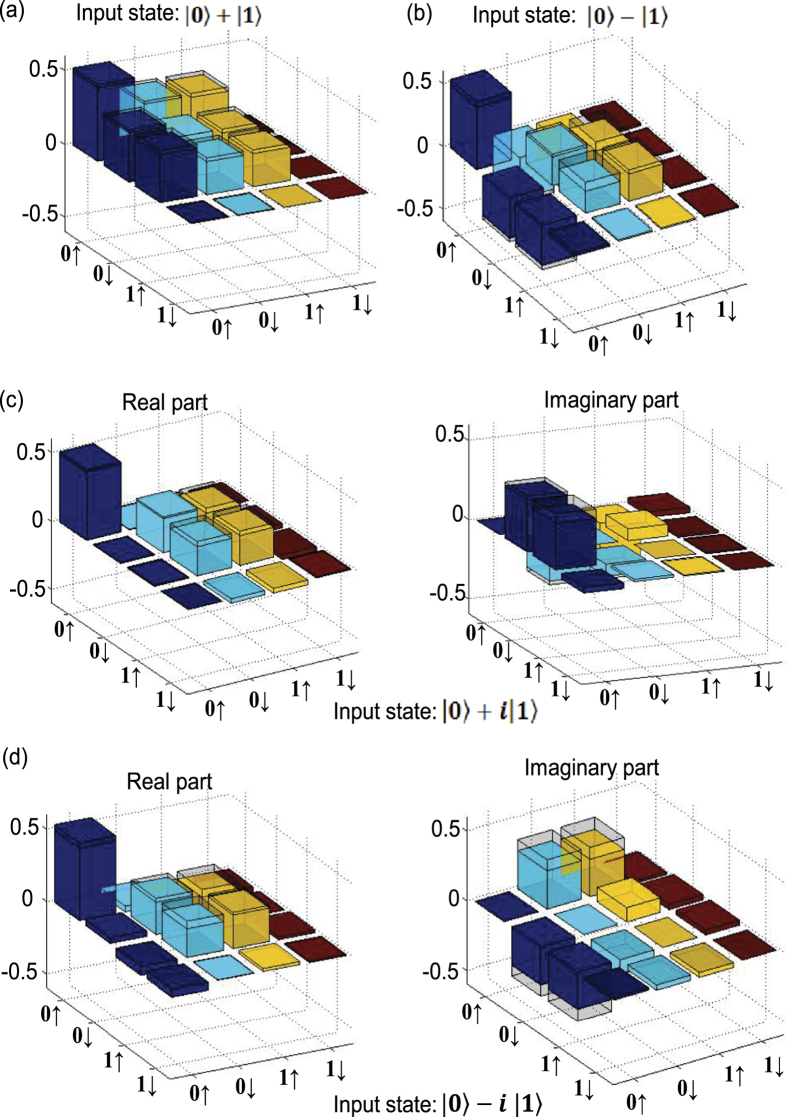
The output state of quantum cloning operation reconstructed through quantum state tomography. The measured density matrix elements of the final state under different input states: (**a**) with input state |0〉 + |1〉; (**b**) with input state |0〉 − |1〉; (**c**) with input state |0〉 + *i*|1〉; (d) with input state |0〉 + *i*|1〉. In (a) and (**b**), we only show the real parts of the density matrix elements and the imaginary parts are small (the largest imaginary matrix element is 0.081*i*). The hollow caps in (**a–d**) denote the matrix elements of the corresponding ideal output state under a perfect quantum cloning operation.

To verify quantum cloning operation, we use quantum state tomography to reconstruct the experimental density matrix *ρ*_*en*_ of the electron and the nuclear spins. Quantum state tomography requires measurements in several complementary bases^37^, and we use a combination of microwave and r.f. pulses to achieve the required rotation of the measurement bases. We show in Fig. 2(a–d) the reconstructed density matrix elements of *ρ*_*en*_ for four complementary input states with *ϕ* = 0, *π*/2, *π*, 3*π*/2, respectively. The results are compared with the corresponding matrix elements of |Φ_*en*_〉〈Φ_*en*_| in the ideal case. The state fidelity *F*_*s*_ = 〈Φ_*en*_|*ρ*_*en*_|Φ_*en*_〉 is defined as the overlap between the real experimental output *ρ*_*en*_ and the ideal output |Φ_*en*_〉. The average state fidelity over these four input states is given by (91.9 ± 0.7)%. The error bar comes from the statistical fluctuation of the photon numbers associated with the optical detection of the spin state and is propagated from the detected quantities to the state fidelity through exact numerical simulation^30^.

**Figure 3:**
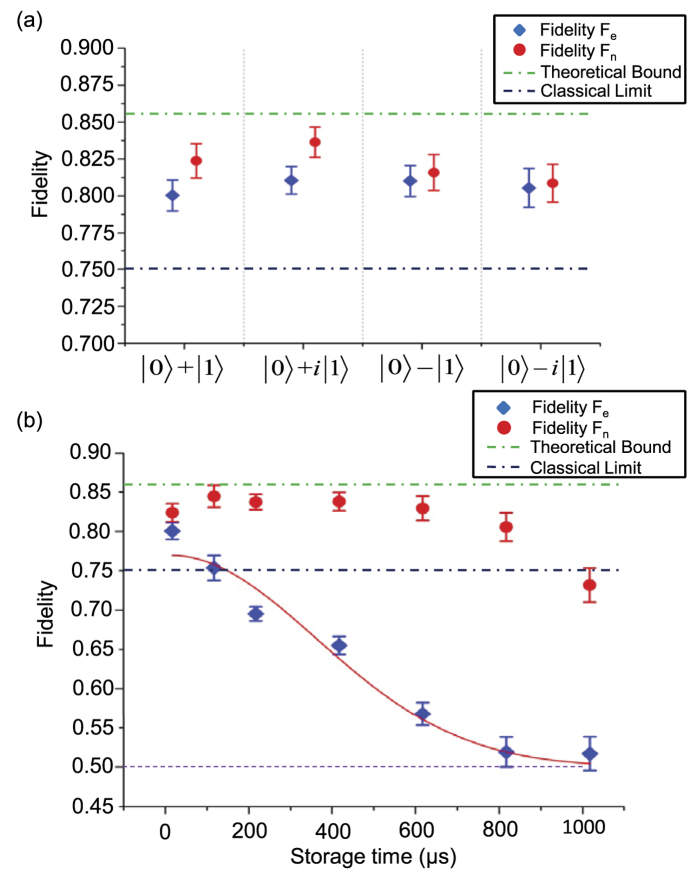
The cloning fidelities and their decay after storage in a quantum memory. (**a**) The measured cloning fidelities for the electron spin and the nuclear spin under different input states. The error bar denotes the standard deviation of measurements. (**b**) The decay of the cloning fidelities *F*_*e*_ and *F*_*n*_ with the input state |0〉 + |1〉 after a tunable storage time. The decay of *F*_*e*_ for the electron spin can be fit by *F*_*e*_ = 0.5 + 0.27*exp*(−*T*^2^/*t*_*d*_) (the red curve), where 0.5 is the lowest value of *F*_*e*_ corresponding to a completely decohered electron spin state, *T* is the total delay time between state preparation and measurement, and *t*_*d*_ ≈ (495 ± 56) *μs* is the dephasing time of the electron spin under the spin echo and strong magnetic field.

From the experimentally reconstructed density matrix *ρ*_*en*_, we calculate the cloning fidelities *F*_*e*_ = 〈Ψ|*tr*_*n*_(*ρ*_*en*_)|Ψ〉 and *F*_*n*_ = 〈Ψ|*tr*_*e*_(*ρ*_*en*_)|Ψ〉 of the reduced states for the electron spin and the nuclear spin, respectively. The results are shown in Fig. 3(a) for the above four complementary input states, with all the fidelities larger than 80%. From these data, the average cloning fidelity is given by 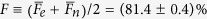. For a classical phase-covariant cloning machine, the optimal average fidelity is bounded from above by *F*_*c*_ ≤ 75% for the above four input states with equal weight (see supplementary information). The experimentally observed fidelity is apparently larger than the classical bound, which unambiguously demonstrates the quantum nature of this cloning machine.

### Demonstration of storage of the cloned quantum state

An important feature of this cloning machine is that the cloned state is stored in the nuclear spin, which is a good quantum memory with coherence time much longer than that of the electron spin. To demonstrate the memory effect, we delay the quantum state tomography measurement after a storage time *T* and examine the decay of the cloning fidelities with the delay time *T*. The result is shown in Fig. 3(b). Even with a Hahn spin echo pulse set at the right position, the fidelity *F*_*e*_ drops to the level of classical bound (75%) after 100 *μs*, while the fidelity *F*_*n*_ in this case remains almost unchanged (the variation is within the error bar). After a much longer storage time of about 1 ms, the fidelity *F*_*n*_ starts to drop to the level of classical bound. Before this time, the fidelity *F*_*e*_ has already decreased to 50%, the lowest value of *F*_*e*_ corresponding to a completely decohered reduced state for the electron spin. In our diamond sample, the coherence time of the nuclear spin is mainly limited by its dipole interaction with the bath nuclear spins and the hyperfine coupling with the electron spin. The coherence time of the nuclear spin qubit can be further increased by several orders of magnitudes with use of the isotopically purified diamond sample with a much dilute bath of nuclear spins^23^,^27^.

In conclusion, we have demonstrated deterministic cloning of quantum states from the electron spin to the nuclear spin in a hybrid spin register with a room-temperature diamond sample. The realization of a deterministic cloning machine, together with the ability to store the cloned quantum states in the nuclear spin quantum memory, provides a key step for application of quantum cloning in quantum information science.

## Additional Information

**How to cite this article**: Wang, W.-B. *et al.* Memory-built-in quantum cloning in a hybrid solid-state spin register. *Sci. Rep.* **5**, 12203; doi: 10.1038/srep12203 (2015).

## Supplementary Material

Supplementary Information
