# Supplementary material for: Memory-built-in quantum cloning in a hybrid solid-state spin register
Source: Sci Rep. 2015 Jul 16;5:12203. doi: 10.1038/srep12203 (PMC4503958; doi:10.1038/srep12203)
Supplement: Supplementary Information [file srep12203-s1.pdf]

Supplementary information: Memory-built-in quantum cloning in a hybrid solid-state spin register

W.-B. Wang[1], C. Zu*[1], L. He[1], W.-G. Zhang[1], L.-M. Duan[2,1]
[1] *Center for Quantum Information, IIIS, Tsinghua University, Beijing 100084, PR China and*
[2] *Department of Physics, University of Michigan, Ann Arbor, Michigan 48109, USA*
(Dated: June 10, 2015)

PACS numbers:

EXPERIMENTAL SETUP

Our experimental setup is described in detail in Ref. [1]. We use a home-built confocal microscopy, with an oil-immersed objective lens, to address and detect single NV center in a type IIa single-crystal synthetic diamond sample from the Element Six company. For each experimental cycle, we start the sequence with 2 μs laser illumination (at 532 nm wavelength) to polarize the NV electron and the nearby nuclear spins (C^{13} and the host N^{14}) and end it with a 3 μs laser pulse for spin state detection. The polarization of the nuclear spins is achieved through the almost degeneracy of the electron spin levels $|m=0\rangle$ and $|m=-1\rangle$ in the optically excited state under an external magnetic field of 427 G, which facilitates electron-spin nuclear-spin flip-flop process during optical pumping [2].

To read out, the spin states are distinguished through their different fluorescence levels under illumination of a short laser pulse, and the fluorescence levels of different spin states are calibrated through the method described in Ref. [1]. We collect signal photons for 0.3 μs right after the detection laser rises to its full intensity, and another 0.3 μs for reference 2 μs later. With a photon collection rate of 80 kHz, we have an average of 0.024 photon counts per cycle. For measurement of each datum, we repeat the experimental cycle at least 6×10^6 times, resulting in a total photon counts of 1.4×10^5. The error bars of our data account for the statistical error associated with the photon detection. We use Monte Carlo simulation to calculate the error bar of each datum, assuming a Poissonian distribution for the photon counts.

ELECTRON SPIN DEPHASING AND SPIN ECHO

One step of our cloning operation is achieved with a r.f. pulse to perform a conditional gate on the nuclear spin. As the gate on the nuclear spin is slow with the gate time longer than the electron spin coherence time under free evolution (about 1.7 μs), we need to consider the influence of dephasing of the electron spin. The dephasing of the electron spin is caused by the hyperfine interaction with a bath of nuclear spins. The effect of the nuclear spin bath on the electron spin can be modelled by an effective magnetic field $B\left(t\right)$, which is slowly fluctuating with time t. Within 100 μs, the time dependence of $B\left(t\right)$ is negligible. So the field B just takes an unknown constant value for each experimental trial, but varies from trial to trial. The influence of the unknown B field needs to be removed in our measurements to verify quantum cloning, and this can be achieved with a single Hahn spin echo pulse, as we show now.

In our implementation, a r.f. $\pi/2$-pulse performs the controlled Hadamard gate CH_{12}. Assume this gate takes time t_g. With influence of the B field taken into account, the state after this gate is $|0,\uparrow\rangle/\sqrt{2}+e^{i(\varphi+Bt_g)}|1\rangle\left(|\uparrow\rangle+|\downarrow\rangle\right)/2$, where we have assumed that the accumulated phase caused by the B field is s_zBt_g (s_z is the electron spin Zeeman number), neglecting the unimportant pre-factor. A microwave π-pulse, which is short compared with $1/B$ and considered to be instantaneous, applies to the transition $|1,\downarrow\rangle\leftrightarrow|0,\downarrow\rangle$ and transfers the state to the form $|0,\uparrow\rangle/\sqrt{2}+e^{i(\varphi+Bt_g)}\left(|0,\downarrow\rangle+|1,\uparrow\rangle\right)/2$. Then, we wait for a time t_1 and applies an instantaneous microwave π-pulse (spin echo pulse) to flip the electron spin $|1\rangle\leftrightarrow|0\rangle$, and the state of the system becomes $|1,\uparrow\rangle/\sqrt{2}+e^{i\varphi}\left(e^{iBt_g}|1,\downarrow\rangle+e^{iB(t_g+t_1)}|0,\uparrow\rangle\right)/2$. After another delay time t_2, we perform the quantum state tomography measurements on the state $|\Phi_f\rangle=e^{iBt_2}|1,\uparrow\rangle/\sqrt{2}+e^{i\varphi}\left(e^{iB(t_g+t_2)}|1,\downarrow\rangle+e^{iB(t_g+t_1)}|0,\uparrow\rangle\right)/2$. Note that quantum state tomography consists of measurements in several complementary bases to map out the density matrix elements of the final state one by one [3]. In implementation of quantum state tomography, we need to apply a combination of r.f. and microwave pulses to rotate the measurement basis, where the r.f. pulse may take another time t_d (note that t_d is basis-dependent). As an example, if we measure the coherence $|1,\uparrow\rangle\langle1,\downarrow|$ between the first two terms of $|\Phi_f\rangle$, we apply a microwave π-pulse (instantaneous) to transfer $|1,\downarrow\rangle$ to $|0,\downarrow\rangle$, a r.f. π-pulse with time t_d to transfer $|1,\uparrow\rangle$ to $|1,\downarrow\rangle$, and a microwave $\pi/2$-pulse to the transition $|1,\downarrow\rangle\leftrightarrow|0,\downarrow\rangle$ to transfer the coherence term

$|1,\uparrow\rangle\langle 1,\downarrow|$ finally to the population difference in $|1\rangle$ and $|0\rangle$ levels, which can be read out through optical detection. With choice of $t_1 = t_2$ and $t_g = t_d$, the effect of the unknown B field is removed. Other coherence terms of the final density matrix are read out by similar methods with an appropriate choice of t_2 and t_d.

PROOF OF THE FIDELITY BOUND FOR CLASSICAL CLONING

The fidelity bound for classical cloning is known to be 2/3 for university quantum cloning where the input state is randomly taken from arbitrary qubit states [4]. The fidelity bound becomes 3/4 for classical phase covariant quantum cloning with the input randomly taken from the states on the equator of the Bloch sphere. Motivated by the connection with the BB84 quantum key distribution protocol, we consider here that the input state is randomly taken from a finite set of states $\left\{\left(|0\rangle + e^{i\varphi}|1\rangle\right)/\sqrt{2}\right\}$ with $\varphi = 0, \pi/2, \pi, 3\pi/2$. We prove that the average fidelity for any classical cloning machine is still upper bounded by 3/4 in this case.

For classical cloning, we first make a measurement on the input state and then prepare two copies of the states based on the measurement information. The best detection strategy is given by a von Neumann measurement in the qubit space [4], which projects the input state $|\Psi\rangle$ to $|v\rangle$ and $|v_\perp\rangle$ with probabilities $|\langle v|\Psi\rangle|^2$ and $|\langle v_\perp|\Psi\rangle|^2$, respectively, where $|v\rangle$ and $|v_\perp\rangle$ denote two arbitrary orthogonal qubit states. When the measurement outcome is $|v\rangle$ (or $|v_\perp\rangle$), we prepare two copies of the outcome state $|v\rangle$ (or $|v_\perp\rangle$), with the cloning fidelity $|\langle v|\Psi\rangle|^2$ (or $|\langle v_\perp|\Psi\rangle|^2$) for this case. The average classical cloning fidelity for the input state $|\Psi\rangle$ is given by

$$F_c = (|\langle v|\Psi\rangle|^4 + |\langle v_\perp|\Psi\rangle|^4). \tag{1}$$

When the input $|\Psi\rangle$ is randomly taken from the set of four states $|\Psi_1\rangle = (|0\rangle + |1\rangle)/\sqrt{2}$, $|\Psi_2\rangle = (|0\rangle - |1\rangle)/\sqrt{2}$, $|\Psi_3\rangle = (|0\rangle + i|1\rangle)/\sqrt{2}$, and $|\Psi_4\rangle = (|0\rangle - i|1\rangle)/\sqrt{2}$ with equal probability $p = 1/4$, the best choice of the measurement basis vectors $|v\rangle$ and $|v_\perp\rangle$ apparently should also be from the states on the equator of the Bloch sphere with $|v\rangle = \left(|0\rangle + e^{i\phi}|1\rangle\right)/\sqrt{2}$ and $|v_\perp\rangle = \left(|0\rangle + e^{i(\phi+\pi)}|1\rangle\right)/\sqrt{2}$, where the optimal ϕ is to be determined. The average classical cloning fidelity is then

$$\begin{aligned} F_c &= \frac{1}{4}\left(\sum_{j=1}^{4}(|\langle v|\Psi_j\rangle|^4 + |\langle v_\perp|\Psi_j\rangle|^4\right) \\ &= \frac{1}{4}\left[\cos^4(\frac{\phi}{2}) + \cos^4(\frac{\phi}{2} - \frac{\pi}{4}) + \cos^4(\frac{\phi}{2} - \frac{\pi}{2}) + \cos^4(\frac{\phi}{2} - \frac{3\pi}{4}) \right. \\ &\quad \left. + \cos^4(\frac{\phi}{2} + \frac{\pi}{2}) + \cos^4(\frac{\phi}{2} + \frac{\pi}{4}) + \cos^4(\frac{\phi}{2}) + \cos^4(\frac{\phi}{2} - \frac{\pi}{4})\right] \\ &= \frac{1}{4}\left[3\cos^4(\frac{\phi}{2}) + 3\sin^4(\frac{\phi}{2}) + 6\cos^2(\frac{\phi}{2})\sin^2(\frac{\phi}{2})\right] = \frac{3}{4}. \end{aligned} \tag{2}$$

The fidelity F_c is actually independent of the choice of parameter ϕ. As F_c is for the optimal classical cloning strategy, we conclude that the average fidelity for any classical cloning machine is always upper bounded by 3/4 when the input is taken from the set of four different states as in the BB84 protocol.

[1] Zu, C. *et al.* Experimental realization of universal geometric quantum gates with solid-state spins. *Nature* 514, 72-75 (2014).

[2] Jacques, V. *et al.* Dynamic polarization of single nuclear spins by optical pumping of nitrogen-vacancy color centers in diamond at room temperature. *Phys. Rev. Lett.* 102, 057403 (2009).

[3] James, D. F. V., Kwiat, P. G., Munro, W. J. & White, A. G. Measurement of qubits. *Phys. Rev. A* **64**, 052312 (2001).

[4] Gisin, N., Ribordy, G., Tittel, W., & Zbinden, H. Quantum cryptography. *Rev. Mod. Phys.* 74, 145-195 (2002).
